# Atrial Fibrillation Dynamics and Ionic Block Effects in Six Heterogeneous Human 3D Virtual Atria with Distinct Repolarization Dynamics

**DOI:** 10.3389/fbioe.2017.00029

**Published:** 2017-05-08

**Authors:** Carlos Sánchez, Alfonso Bueno-Orovio, Esther Pueyo, Blanca Rodríguez

**Affiliations:** ^1^Biosignal Interpretation and Computational Simulation (BSICoS), I3A and IIS, University of Zaragoza, Zaragoza, Spain; ^2^Defense University Centre (CUD), General Military Academy of Zaragoza (AGM), Zaragoza, Spain; ^3^Department of Computer Science, University of Oxford, Oxford, UK; ^4^Biomedical Research Networking Center in Bioengineering, Biomaterials and Nanomedicine (CIBER-BBN), Zaragoza, Spain

**Keywords:** arrhythmia, human atria, electrophysiology, variability, model populations, electrical propagation, fibrillatory patterns

## Abstract

Atrial fibrillation (AF) usually manifests as reentrant circuits propagating through the whole atria creating chaotic activation patterns. Little is yet known about how differences in electrophysiological and ionic properties between patients modulate reentrant patterns in AF. The goal of this study is to quantify how variability in action potential duration (APD) at different stages of repolarization determines AF dynamics and their modulation by ionic block using a set of virtual whole-atria human models. Six human whole-atria models are constructed based on the same anatomical structure and fiber orientation, but with different electrophysiological phenotypes. Membrane kinetics for each whole-atria model are selected with distinct APD characteristics at 20, 50, and 90% repolarization, from an experimentally calibrated population of human atrial action potential models, including AF remodeling and acetylcholine parasympathetic effects. Our simulations show that in all whole-atria models, reentrant circuits tend to organize around the pulmonary veins and the right atrial appendage, thus leading to higher dominant frequency (DF) and more organized activation in the left atrium than in the right atrium. Differences in APD in all phases of repolarization (not only APD_90_) yielded quantitative differences in fibrillation patterns with long APD associated with slower and more regular dynamics. Long APD_50_ and APD_20_ were associated with increased interatrial conduction block and interatrial differences in DF and organization index, creating reentry instability and self-termination in some cases. Specific inhibitions of I_K1_, I_NaK_, or I_Na_ reduce DF and organization of the arrhythmia by enlarging wave meandering, reducing the number of secondary wavelets, and promoting interatrial block in all six virtual patients, especially for the phenotypes with short APD at 20, 50, and/or 90% repolarization. This suggests that therapies aiming at prolonging the early phase of repolarization might constitute effective antiarrhythmic strategies for the pharmacological management of AF. In summary, simulations report significant differences in atrial fibrillatory dynamics resulting from differences in APD at all phases of repolarization.

## Introduction

Reentrant circuits are one of the main manifestations of the most commonly diagnosed arrhythmia in the human heart: atrial fibrillation (AF) (Fuster et al., 2011). Chronic AF causes electrical remodeling in cardiac tissue, resulting in short refractory periods and slow conduction favoring reentries (Nattel et al., 2008), as well as structural remodeling, including fibrosis that alters atrial tissue composition (Burstein and Nattel, 2008; Harrison et al., 2015; Miragoli and Glukhov, 2015). AF frequently arises by rapid ectopic activity around the pulmonary veins (PV) generating stable reentrant circuits combined with short duration wavelets that propagate through the whole atria (Nattel et al., 2008). Reentrant sources are usually generated in the left atrium (LA) with propagation of the fibrillatory waves to the right atrium (RA) (Haïssaguerre et al., 1998). Its underlying mechanisms are not completely understood yet due to the complexity of teasing out structural and electrophysiological contributors (Burstein and Nattel, 2008; Nattel et al., 2008; Harrison et al., 2015; Miragoli and Glukhov, 2015).

Current anti-AF therapies aim at decreasing heart rate, blocking reentrant pathways, and isolating ectopic foci [normally ablation combined with antiarrhythmic drugs (Ames and Stevenson, 2006)]. Their efficacy is, however, limited (Jais and Packer, 2007). Further improvements in the management of AF will be facilitated by a better characterization of atrial dynamics and electrophysiological substrates through the analysis of specific measurable indices. As an example, the frequency of tissue activation or the organization in atrial activation can be quantified from electrograms (EGMs) measured on the atrial surface. Heterogeneity in these indices is considered putative of the complex spatiotemporal patterns that characterize AF, including multiple self-sustained rotors driving fibrillatory conduction (Berenfeld and Jalife, 2011; Atienza et al., 2015; Benharash et al., 2015), collision of the resulting wavefronts (Narayan et al., 2011), and disorganized local activation due to rotor meandering (Zlochiver et al., 2008; Berenfeld and Jalife, 2011). Importantly, all these measurable indices (and hence AF characteristics) present significant variability between different patients, but the implications of this variability are not well understood (Habel et al., 2010; Britton et al., 2013; Sánchez et al., 2014; Kogawa et al., 2015; Muszkiewicz et al., 2016).

In this study, we investigate AF dynamics in virtual patients exhibiting intersubject variability in atrial electrophysiological properties and differences in their response to ionic block used for antiarrhythmic therapy. In order to isolate the contribution of the electrophysiological properties from the anatomical substrate, we recreated these patients by constructing realistic 3D human atrial models with different action potential duration (APD) properties but with the same anatomy and structure (Seemann et al., 2006). Rotor dynamics for each virtual patient were analyzed using EGM maps distributed through the whole atria and validated through comparison against clinical EGM recordings. Specifically, we quantified how key intersubject differences in the repolarization of the action potential (AP) affected atrial activation patterns in AF. The potential effect of antiarrhythmic strategies on each virtual patient was assessed through the simulation of specific ionic blocks.

## Materials and Methods

### Construction of Six Human Whole-Atria Electrophysiology Models

The human whole-atria anatomical model described in Seemann et al. (2006) was used as the basis to construct the six virtual human patient models of AF. The whole-atria anatomical model includes fiber orientation, conduction anisotropy, and spatial heterogeneities in ionic currents and conduction velocity (CV) in the main atrial structures: LA, RA, sinoatrial node (SAN), cresta terminalis, pectinate muscles, fossa ovalis, Bachmann’s bundle, cavotricuspid isthmus, left atrial appendage (LAPG), right atrial appendage (RAPG), atrioventricular ring, and interatrial bridges (Seemann et al., 2006; Krueger et al., 2011; Tobon et al., 2013). Anisotropic ratio (transversal to longitudinal ratio of conductivity) and spatial heterogeneities are summarized in Table S1 in Supplementary Material.

**Figure 1:**
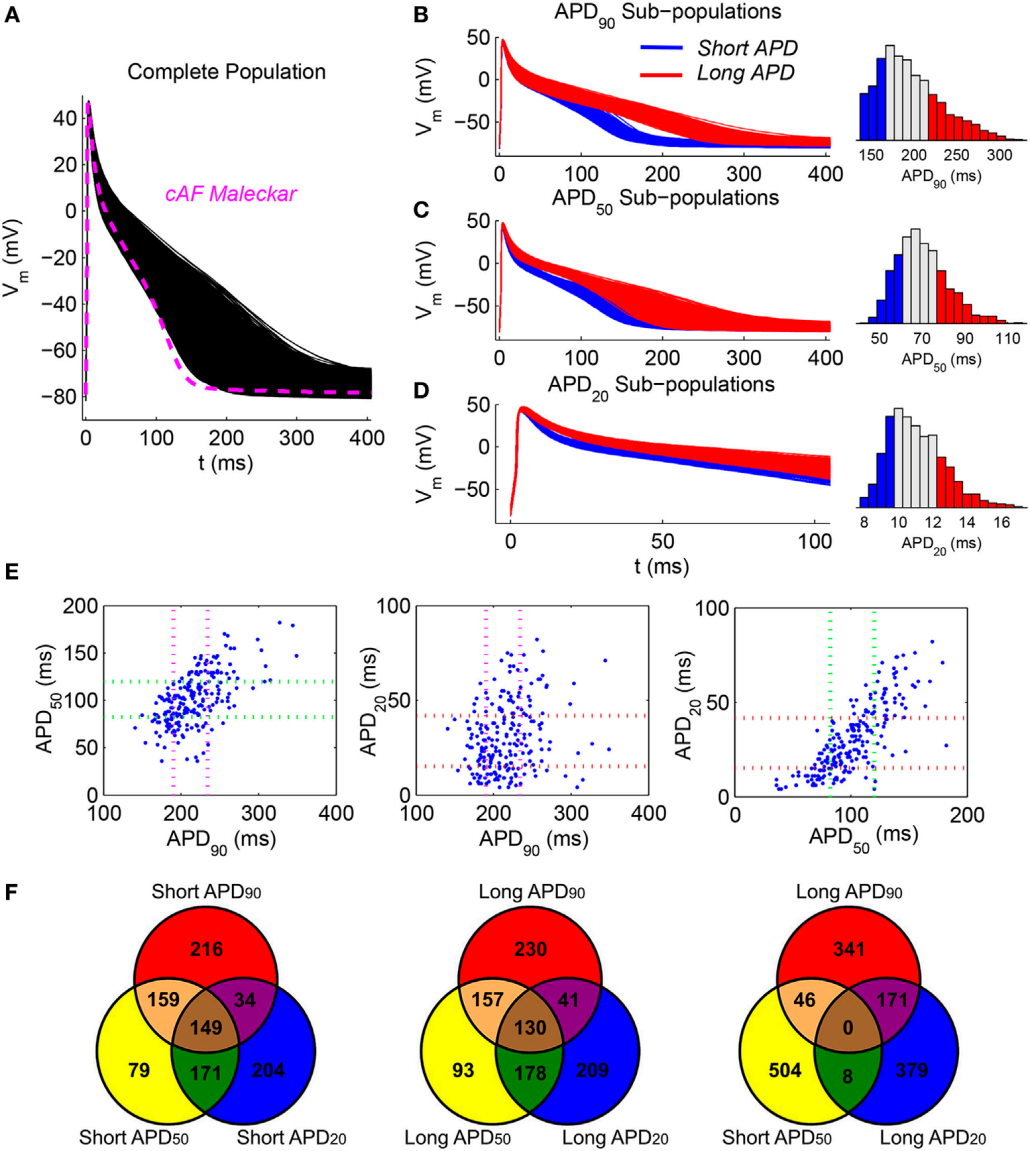
**Considered atrial fibrillation (AF) phenotypes based on cellular repolarization characteristics**. **(A)** Population of simulated AF cell models calibrated against experimental recordings (black). The baseline Maleckar et al. model after chronic-AF remodeling (magenta) is provided for comparison. **(B–D)** First (blue) and fourth (red) quartile subpopulations according to APD_90_, APD_50_, and APD_20_, respectively, together with the corresponding histograms. Note magnification in time axis in panel **(D)**. **(E)** Scatter plots of experimentally measured APD_90_, APD_50_, and APD_20_. Dotted lines represent first and third quartile values for each group of experimental action potential durations (APDs). **(F)** Venn diagrams with the number of action potential (APs) belonging to one, two, or three subpopulations of models for different intersections of short and long APD phenotypes.

Each human whole-atria model was assigned a different AP phenotype with human atrial membrane kinetics based on the Maleckar et al. model (Maleckar et al., 2009), but with ionic conductances calibrated to account for AF remodeling and intersubject variability in APD using an experimentally calibrated population of human AP models as described in Sánchez et al. (2014). In short, a population of 2,275 AP atrial models was constructed based on an AF-remodeled version of the Maleckar et al. model (70% decrease in I_CaL_, 50% decrease in I_to_, 50% decrease in I_Kur_, and 100% increase in I_K1_), considering variability in six ionic current conductances (I_K1_, I_NaK_, I_CaL_, I_to_, I_Kur_, and I_NaCa_) and calibrated using AP recordings from human atrial trabeculae (Wettwer et al., 2004, 2013). In order to evaluate effects of differences in APD_90_, APD_50_, or APD_20_ values on atrial dynamics, the human AP models (Figure 1A) were classified into six subpopulations based on whether they exhibited long or short APD_90_, APD_50_, or APD_20_ values (Figures 1B–D). For each degree of repolarization (APD_90_, APD_50_, or APD_20_), the human atrial models yielding APD within the first quartile in the overall population were classified as “short APD,” and the models above the third quartile as “long APD.” For example, the short APD_90_ subpopulation included all models with APD_90_ shorter than 172.6 ms and the long APD_90_ subpopulation those with APD_90_ longer than 222.3 ms (first and third APD_90_ quartiles, respectively, Figure 1B).

In human atrial cardiomyocytes, APD_90_, APD_50_, and APD_20_ are frequently (although not always) related with each other, i.e., long APD_90_ is in general associated with long APD_50_ and long APD_20_, as shown in Figure 1E *via* scatter plots of experimentally measured APD_90_ vs APD_50_, APD_90_ vs APD_20_, and APD_50_ vs APD_20_. The scatter plots of Figure 1E further illustrate natural overlaps between short/long APD categories (e.g., long APD_90_ vs short APD_50_; Figure 1E, left panel) and less likely intersections (e.g., long APD_20_ vs short APD_50_; Figure 1E, right panel). Such overlaps are also captured in the six generated model subpopulations as illustrated in the Venn diagrams displayed in Figure 1F, showing frequency of intersections in number of APs between the different (and more largely sampled) APD subpopulations.

**Figure 2:**
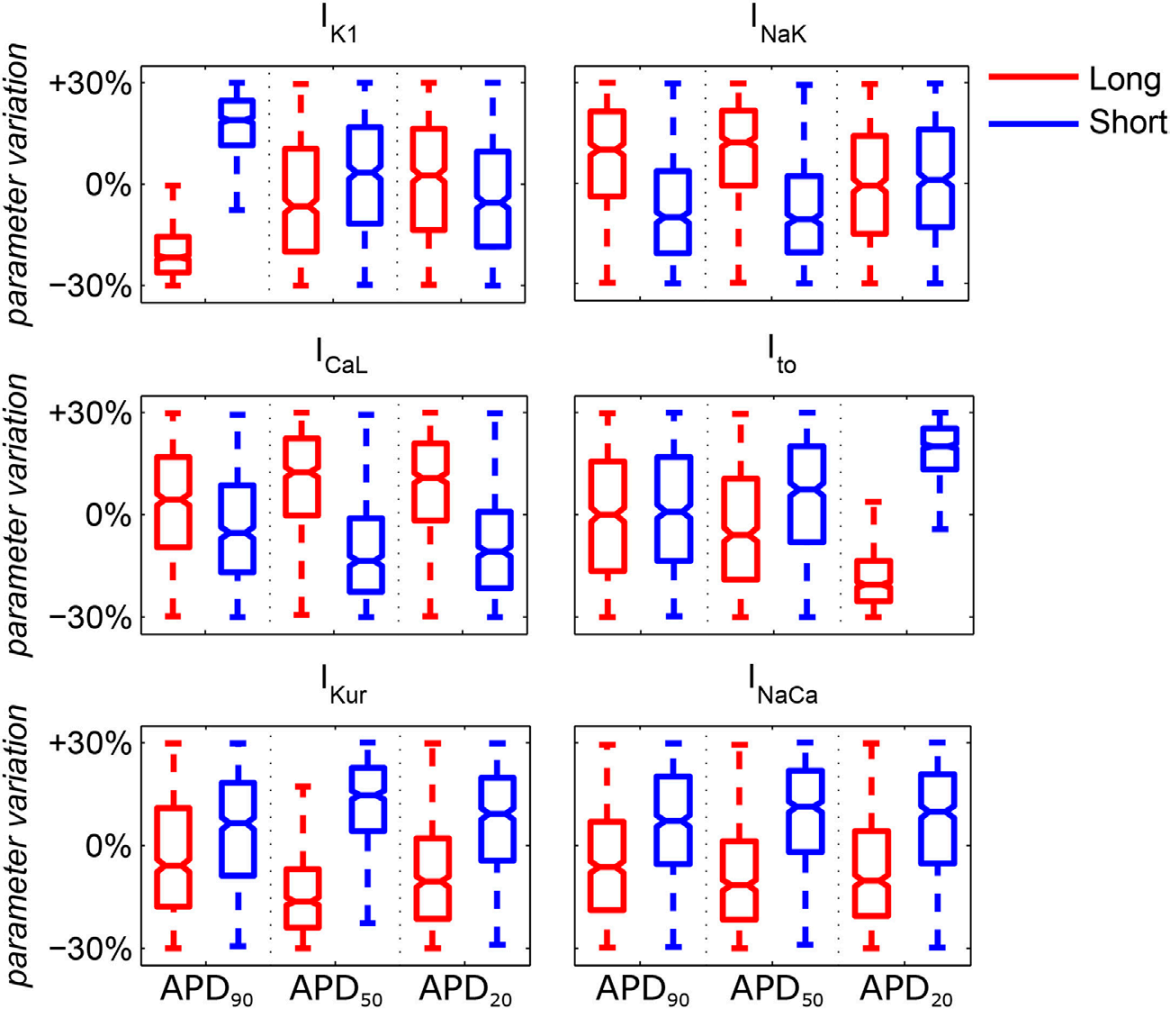
**Median values and ranges of variability in ionic conductances in the six subpopulations of human atrial action potential models under atrial fibrillation remodeling**. Blue and red indicates short and long APD_90_, APD_50_, and APD_20_, respectively. Each boxplot represents the range covered by the ionic conductances: the edges of the box are the first and third quartiles, whiskers extend to the most extreme data points, the estimated median physiological value is the central horizontal line and the notch around the median is the 5% significance level.

The distributions of ionic current conductances in each of the short/long APD_90_, APD_50_, and APD_20_ subpopulations are shown in Figure 2. This is in agreement with experimental studies showing variability in cellular electrophysiology in human cardiomyocytes and, specifically, in the atria (Wang et al., 1993). Each subpopulation of human AP models was used to assign membrane kinetics in one of the six human whole-atria models, by randomly assigning an AP model of the corresponding subpopulation to each cell (once per whole-atrial model). Figure S1 in Supplementary Material shows APD distributions in the six whole-atria models in sinus rhythm. These distributions indicate that the effect of APD heterogeneity due to the existence of different atrial structures has a stronger impact on APD dispersion than the random APD variability within each subpopulation. This means that tissue coupling and macrostructural regional differences in the anatomical model (Table S1 in Supplementary Material) attenuate cell-to-cell differences in APD due to random sampling from the AP subpopulations.

Our simulations also incorporate the effect of a concentration of 1 nM acetylcholine ([ACh]) in the whole models, simulated as in previous studies (Kneller et al., 2002), in order to facilitate the inducibility of arrhythmic behavior (Pandit et al., 2011) and to incorporate electrophysiological heterogeneities in the different atrial structures (Table S1 in Supplementary Material). Given the uncertainty on exact distributions of ACh release in the human atria (Kneller et al., 2002; Jones et al., 2012), we adopted a simplified approach for its modeling, accounting for left and right atrial (LA/RA) differences on I_KACh_ as reported in Sarmast et al. (2003). Larger ACh concentrations yielded an overall reduction in the regularity indices described below, with similar spatial distributions (Figure S2 in Supplementary Material).

### Stimulation Protocol

A preliminary conditioning of each whole-atria model was performed by applying a train of 40 periodic stimuli at the SAN site at a cycle length of 500 ms. Then, six extra-stimuli were applied near the right pulmonary veins (RPV) in the LA to generate reentries and fibrillatory behavior. The cycle length of these six extra-stimuli was different for each AP phenotype (in the range 130–180 ms). All externally applied stimuli were 2-ms duration and twice diastolic threshold current pulses.

### Simulated Ionic Current Reduction

In order to investigate how APD differences may modulate the response to antiarrhythmic therapies, the effects of ionic current block on fibrillatory dynamics and reentrant properties were quantified. Given their importance in atrial dynamics, as identified in previous studies (Kneller et al., 2005; Pandit et al., 2005, 2011; Sánchez et al., 2012), 30% I_K1_ block, 30% I_NaK_ block, and 15% I_Na_ block were simulated in all six whole-atrial models. These degrees of block allow the analysis of fibrillatory properties without critical alterations in cellular electrophysiology and/or electrical propagation (Sánchez et al., 2012), in agreement with experimental studies on the inhibition of I_K1_ with barium (Wu et al., 1999), I_Na_ with flecainide (Barekatain and Razavi, 2012), and I_NaK_ with digitalis (Wasserstrom and Aistrup, 2005). Furthermore, similar degrees of block have been widely used in sensitivity analysis studies (Romero et al., 2009, 2011; Keller et al., 2010; Sánchez et al., 2012; Chang et al., 2015). Simulation of these ionic blocks was initiated 3 s after arrhythmia generation in each of the six AF virtual models.

### Whole-Atria Model Evaluation Based on EGMs of AF Patients

The EGM indices obtained from the six human whole-atria models were compared to intracardiac recordings from AF patients included in the Ann Arbor database (Ann Arbor EGM Libraries, Chicago, IL, USA) (see Figures S3 and S4 in Supplementary Material). We considered only the unipolar EGMs registered on the atrial surface, i.e., those in the high RA and the RAPG. The sampling rate for signal acquisition was 1,000 Hz. The signal processing techniques described in the following section and the Supplementary Material for the simulated EGMs were also applied to calculate dominant frequency (DF), organization index (OI), and regularity index (RI) in the EGM database. Further details in metrics calculation are provided in the Supplementary Material. Coupling index (CP) was not calculated as only one EGM signal per patient was available (Faes and Ravelli, 2007).

In the six virtual models, EGMs were obtained in 49 electrodes covering both the LA and the RA and located at a distance of about 2 mm from the endocardial surface, as in unipolar EGM recordings (Plonsey and Rudy, 1980; Gima and Rudy, 2002; Baher et al., 2007). A fourth-order Butterworth band-pass-filter (*f*_c1_ = 40 Hz; *f*_c2_ = 250 Hz), followed by a signal-rectifier and a fourth-order Butterworth high-pass-filter (*f*_c_ = 20 Hz), were applied to the EGMs in order to minimize spectral components different from those of the main activation wavefront (Botteron and Smith, 1995). The four indices mentioned in the previous paragraph, DF, OI, RI, and CP, were extracted from the 49 EGM signals and interpolated to the whole-atrial tissue for representation.

### Computational Tools

Whole-atrial simulations were conducted using the ELVIRA code, which solves the monodomain equation using the finite element method as described in Heidenreich et al. (2010), with a time step of 0.04 ms ensuring numerical convergence of the results (Figure S5 in Supplementary Material) and minimizing computation time. Signal processing techniques were implemented using Matlab.

## Results

### Modulation of AF Dynamics by Electrophysiological Phenotypic Differences

**Figure 3:**
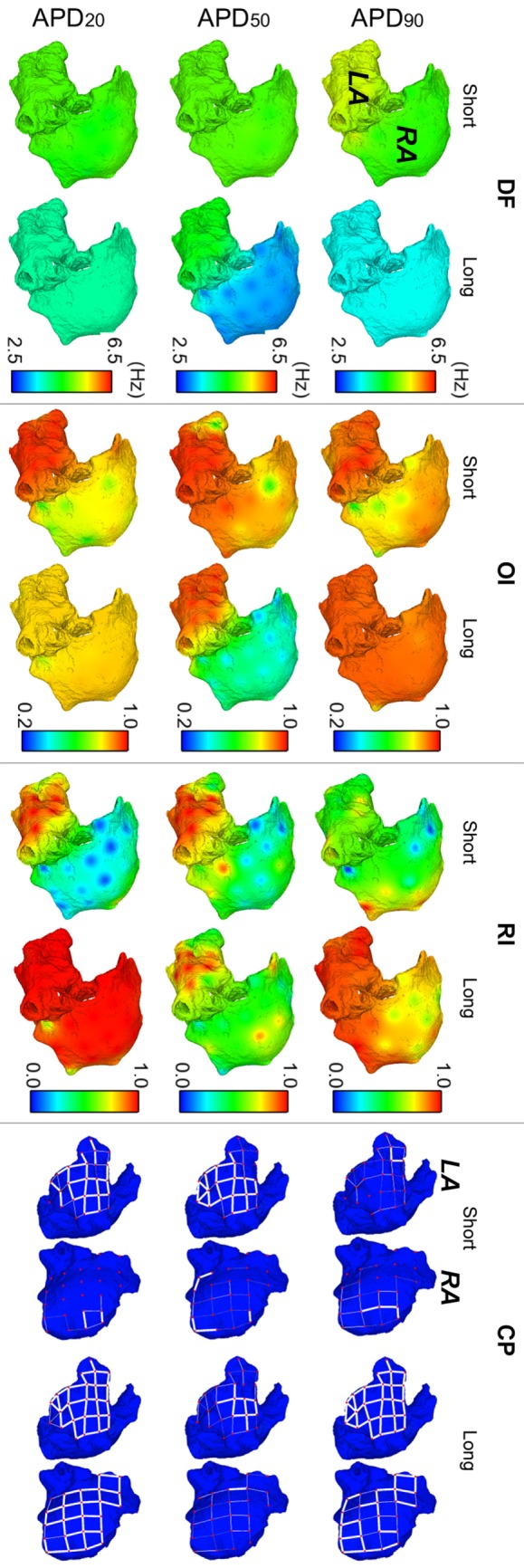
**Modulation of atrial fibrillation reentrant dynamics by intersubject variability in action potential phenotype**. Interpolated maps show dorsal views of the atria for dominant frequency (DF), organization index (OI), regularity index (RI), and coupling index (CP) between adjacent electrogram (thick white lines represent CP > 0.9, thin solid lines represent 0.75 ≤ CP < 0.9, and soft gray lines represent 0.6 ≤ CP < 0.75. CP < 0.6 is represented by absence of lines) for short and long APD_90_, APD_50_, and APD_20_ virtual phenotypes.

Figure 3 illustrates the strong effect of differences in APD in the dynamics of human AF through quantification of DF, RI, and OI in the six virtual patients. AF dynamics were modulated by differences in all three phases of repolarization, as shown in Figure 3 for the models with short vs long APD_90_, APD_50_, and APD_20_. Furthermore, the computed EGMs in the six whole-atria models yielded all arrhythmia-related indices in agreement with those in the Ann Arbor EGM database, as shown in the Supplementary Material.

Fibrillatory dynamics in the human long APD_90_ whole-atria model was sustained by a single stable rotor in the RA, with slower DF than in the short APD_90_ atrial model (top-left panel in Figure 3). The short APD_90_ model exhibited more chaotic AF dynamics (sustained by several reentrant rotors) with high DF values, particularly in the LA and low RI (reflecting changes in activation patterns in different cycles). OI was generally high for both phenotypes, meaning that reentrant rotors presented no frequential components significantly different from DF and its harmonics.

The human model with long APD_50_ exhibited smaller values of DF (1.77 Hz lower in average) and OI (0.42 smaller in average) in the RA than the model with short APD_50_ (second row-left panels in Figure 3). The main reason was a block of interatrial propagation occurring in one out of three wavefronts from the LA to the RA for the long APD_50_ simulation. However, RI and CP were similar for both human models, suggesting local morphological changes of similar magnitude in the EGMs over time and between neighboring regions (second row-right panels in Figure 3).

Interestingly, AF dynamics in human atrial models with short vs long APD_20_ displayed notable differences in fibrillatory patterns. The human atrial long APD_20_ model was associated with very organized and regular activation patterns (OI and RI indices) as compared with those in the human atrial short APD_20_ model. AF dynamics in the short APD_20_ model were similar to those in the short APD_50_ model but with a more chaotic propagation in the RA due to the wavefront meandering, as shown by the lower values of both OI and RI (second and third rows, middle panels in Figure 3). Regarding the long APD_20_, there was only one main rotor, with low DF, high OI, high RI, and high CP in all the tissue (third row in Figure 3), which stopped propagating after 6.3 s of simulation due to interatrial conduction block.

### Modulation of Fibrillatory Behavior in the Six Models by Ionic Blocks

**Figure 4:**
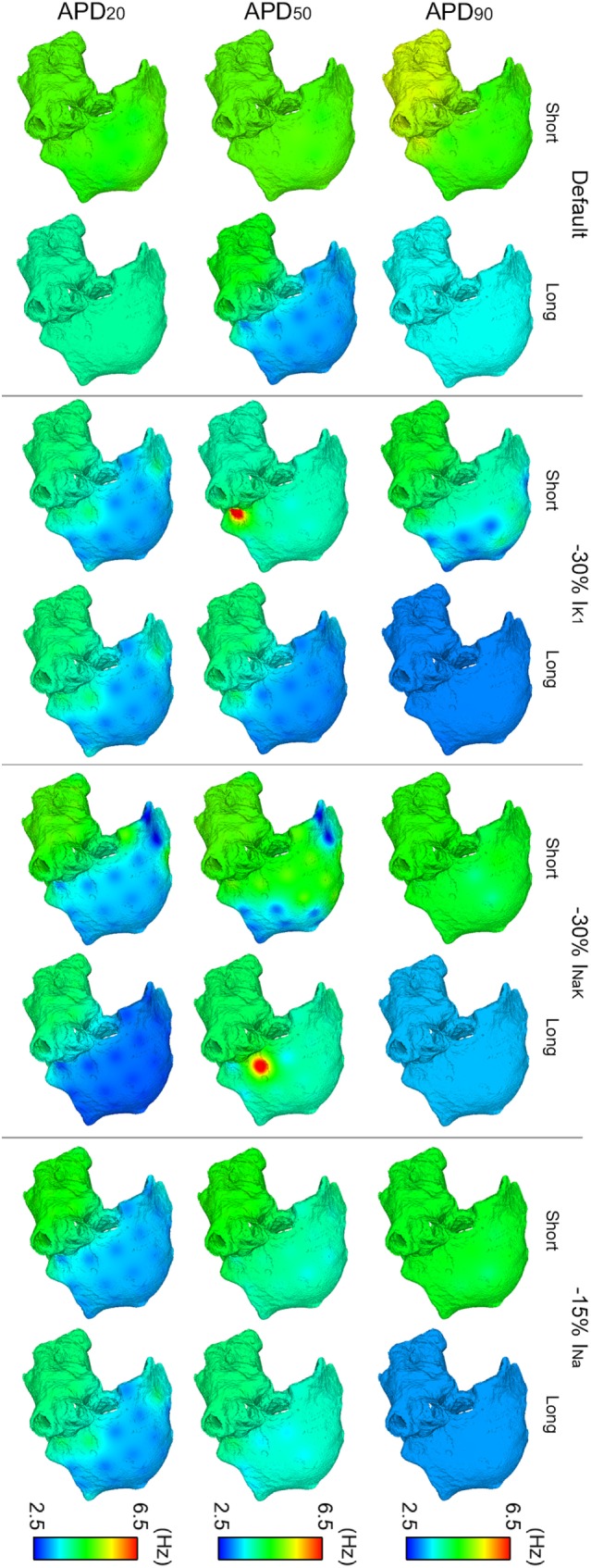
**ContinuedModulation of dominant frequency (DF) of reentry in atrial fibrillation by intersubject variability in action potential phenotype in response to ion channel block**. Interpolated DF maps (dorsal view of the atria) are shown for short and long APD_90_, APD_50_, and APD_20_ subpopulations of models before (default) and after 30% I_K1_ inhibition, 30% I_NaK_ inhibition, and 15% I_Na_ inhibition, respectively.

In spite of differences in atrial dynamics shown in Figure 3, ionic current block yielded similar propagation patterns in all six virtual whole-atria models, as illustrated in Figure 4. The cores of the main reentrant rotors were usually located near the RPV, the LPV, the LAPG, and the RAPG, and secondary rotors usually appeared close to the SVC and the MV. Some particular cases, such as I_K1_ block in the short APD_50_ virtual phenotype as well as I_NaK_ block in both short and long APD_50_ virtual phenotypes, presented significant wave meandering and temporary rotors in the RA. In 17 out of the 18 considered interventions, the fibrillatory behavior remained self-sustained for the duration of the simulation. Only 30% I_NaK_ block in the long APD_90_ virtual model led to arrhythmia termination. Complete information about the number of rotors and reentrant circuits, i.e., those without phase singularities but rotating around an anatomical structure, in the LA and the RA are displayed in Table S2 in Supplementary Material classified by duration and path length for each simulation. The antiarrhythmic effect of the ionic current blocks simulated was, therefore, limited in the simulations, even though the complexity of the arrhythmia was decreased.

Overall, I_K1_, I_NaK_, or I_Na_ block led to smaller DF and more uniform DF distributions in the six virtual phenotypes (Figure 4). This was due to the predominance of the main rotors over secondary rotors entailing additional periodicity of atrial tissue activation. However, significant LA–RA gradients in DF arose in some scenarios due to interatrial propagation block. This was the case in simulations with I_NaK_ and I_Na_ blocks in the short APD_20_ virtual phenotype (third row, right panels in Figure 4). Both I_K1_ block in the short APD_50_ phenotype and I_NaK_ block in the long APD_50_ phenotype entailed low DF in most atrial tissue except for high DF in reduced areas close to the junctions between the LA and the RA (second row, middle panels in Figure 4). This regional high DF was produced by a different delay in the wavefronts propagating and reentering in each atrium due to the APD alterations derived from the ionic blocks. Two depolarizations per rotor cycle were observed in the two cases: one due to the propagation wavefront rotating in the RA and the other due to the income of an earlier wavefront from the LA, colliding afterward with the RA wavefront. These areas did not have time to repolarize completely after their activation by each wavefront and, therefore, depolarizations were locally less prominent.

**Figure 5:**
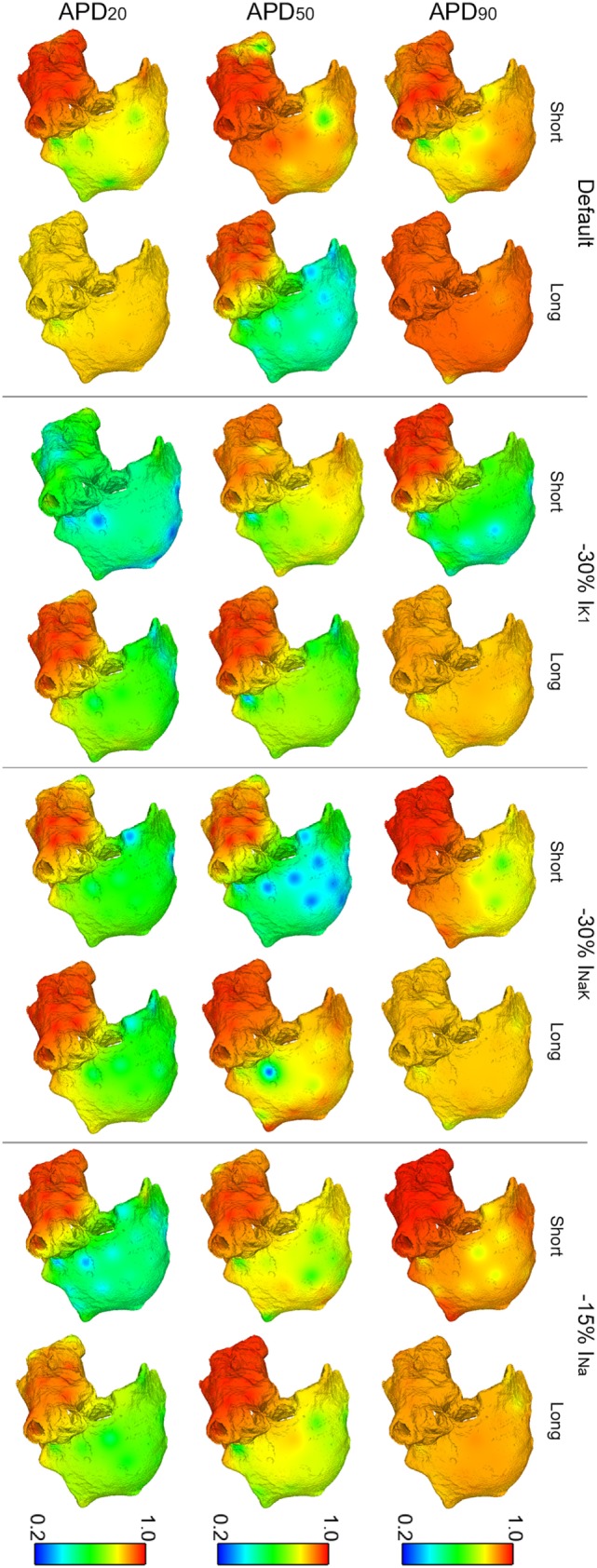
**Modulation of organization index (OI) of reentry in atrial fibrillation by intersubject variability in action potential phenotype in response to ion channel block**. Interpolated OI maps (dorsal view of the atria) are shown for short and long APD_90_, APD_50_, and APD_20_ subpopulations of models before (default) and after 30% I_K1_ inhibition, 30% I_NaK_ inhibition, and 15% I_Na_ inhibition, respectively.

Regarding OI distributions, OI was higher in the LA than in the RA in all the simulated conditions due to the wave meandering and generation of secondary wavelets in the RA. This, thus, led to more disorganized activation patterns in RA than in LA (Figure 5). A significant reduction of OI in the LA was only observed after inhibition of I_K1_ in the short APD_20_ phenotype (third row in Figure 5). The highest OI values were obtained when I_K1_, I_NaK_, or I_Na_ were inhibited in the long APD_90_ phenotype, although these were slightly smaller than those in the phenotype when no ionic blocks were simulated (first row in Figure 5).

**Figure 6:**
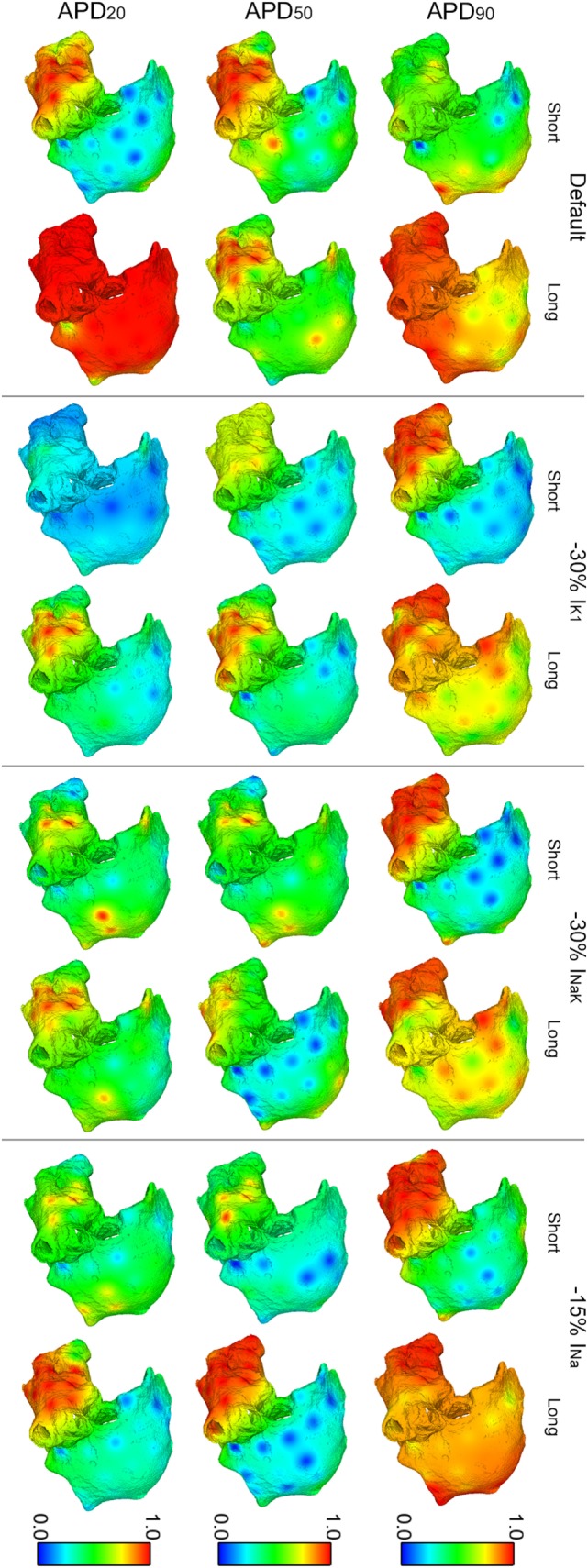
**Modulation of regularity index (RI) of reentry in atrial fibrillation by intersubject variability in action potential phenotype in response to ion channel block**. Interpolated RI maps (dorsal view of the atria) are shown for short and long APD_90_, APD_50_, and APD_20_ subpopulations of models before (default) and after 30% I_K1_ inhibition, 30% I_NaK_ inhibition, and 15% I_Na_ inhibition, respectively.

**Figure 7:**
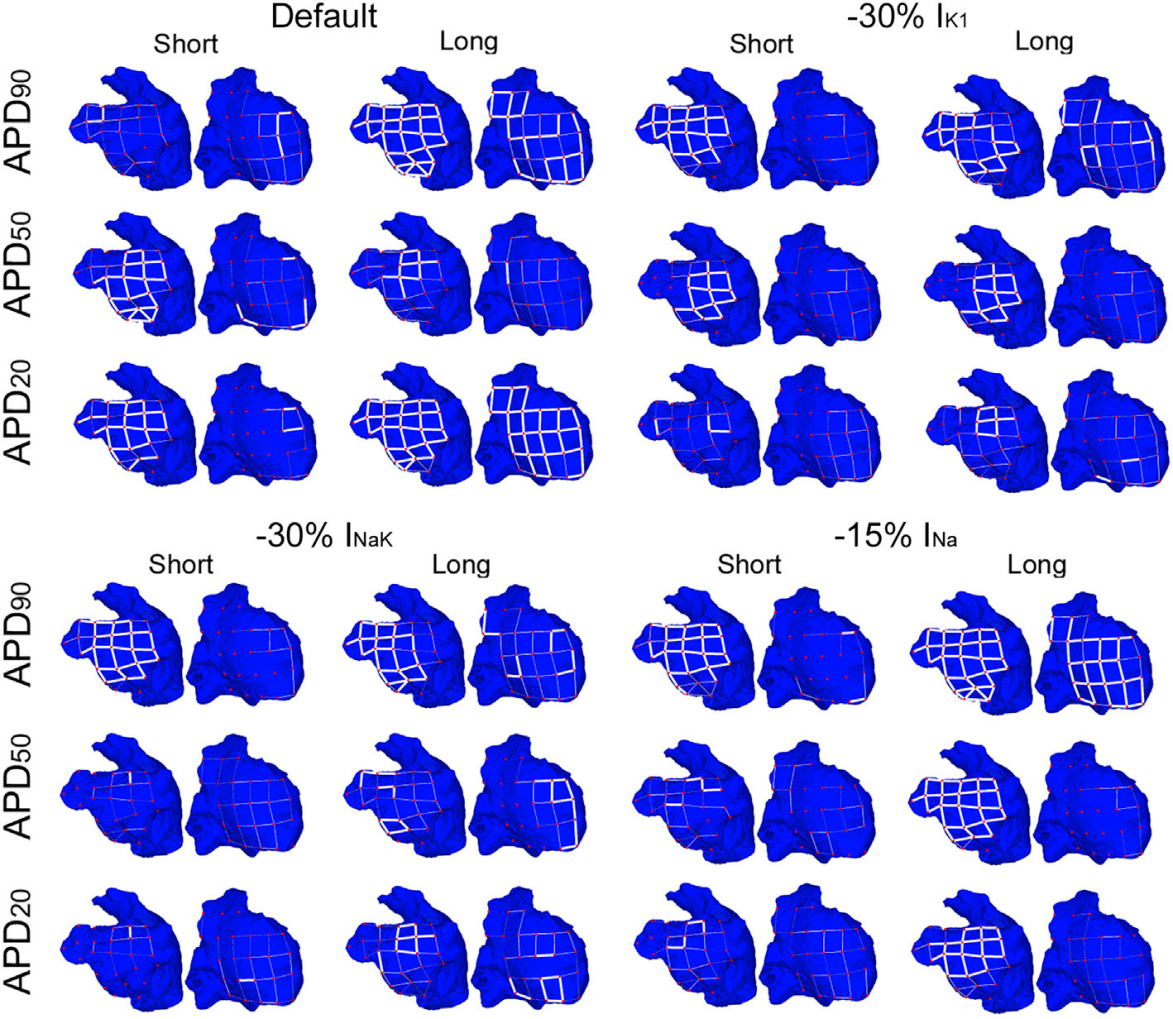
**Modulation of coupling index (CP) of reentry in atrial fibrillation by intersubject variability in action potential phenotype in response to ion channel block**. Dorsal views of the left atrium (left) and right atrium (right) are shown. CP between electrogram in adjacent electrodes (thick white lines represent CP > 0.9, thin solid lines represent 0.75 ≤ CP < 0.9, and soft gray lines represent 0.6 ≤ CP < 0.75. CP < 0.6 is represented by absence of lines) for short and long APD_90_, APD_50_, and APD_20_ subpopulations of models before (default) and after 30% I_K1_ inhibition, 30% I_NaK_ inhibition, and 15% I_Na_ inhibition, respectively.

Figures 6 and 7 show results of RI distributions and CP between adjacent electrodes, respectively. RI values after ionic inhibition were all smaller than in their respective default cases. Furthermore, they were generally higher in the LA than in the RA, highlighting important LA–RA gradients in most of the simulated cases because of the presence of RA rotors with acute meandering, whereas LA activation was usually more stable. As for OI, inhibition of I_K1_ in the short APD_20_ phenotype led to very irregular activation patterns, i.e., low RI, in both atria (third row, second column in Figure 6). The most regular activation patterns were obtained following I_Na_ block in the long APD_90_ phenotype (top-right panel in Figure 6). Regarding CP, a decrease in coupling with respect to the default cases was generally observed after the simulations of ionic blocks (Figure 7). The strongest couplings were observed in the LA after inhibiting I_Na_ in four of the six virtual phenotypes, whereas they were very weak in both atria for the short APD_50_ and APD_20_ phenotypes after blocks of any of the three ionic currents (Figure 7).

**Figure 8:**
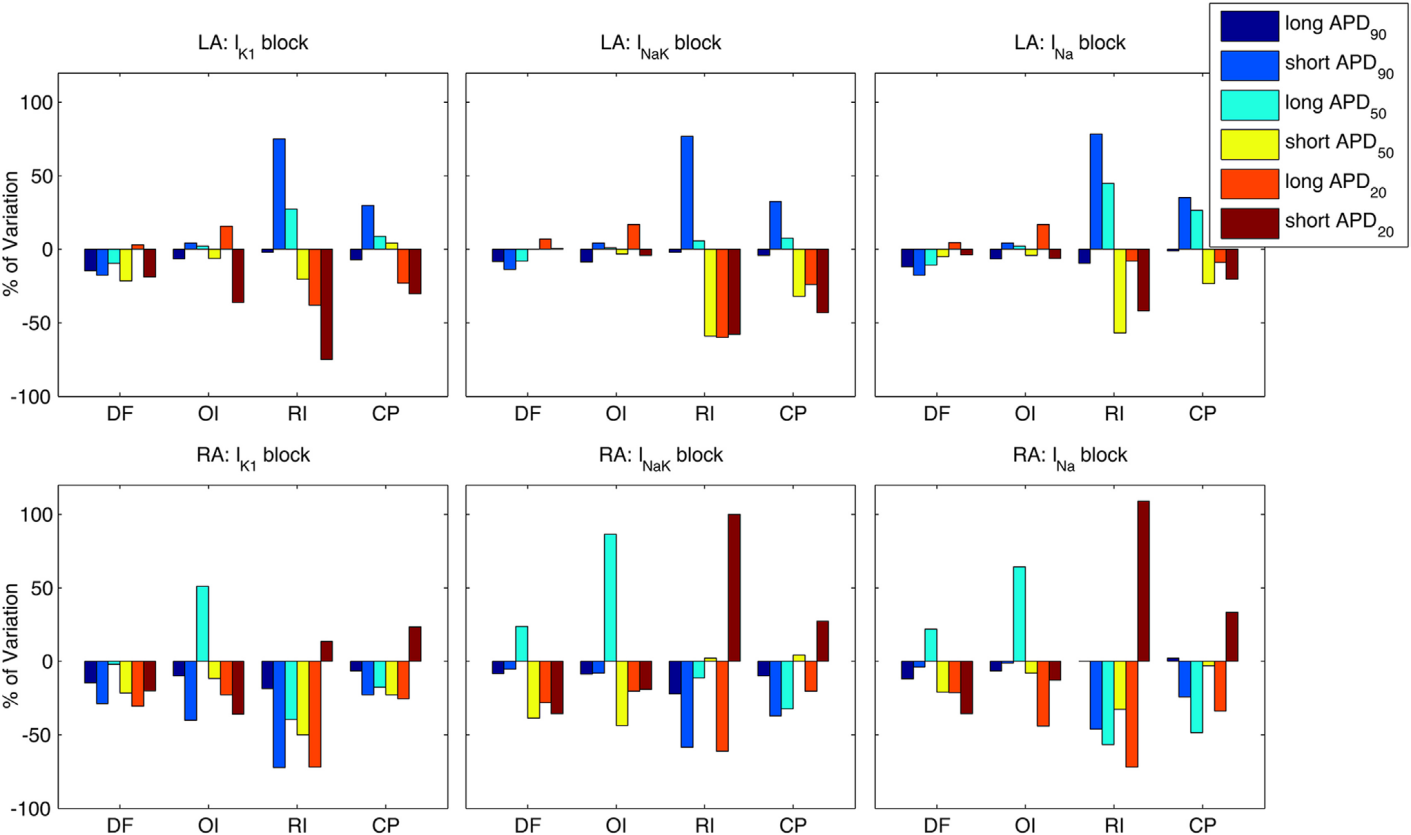
**Modulation by action potential (AP) phenotype in intra-subject and intersubject variability in response to drug block**. Percentages of variation of average indices of atrial fibrillation reentrant dynamics [dominant frequency (DF), organization index (OI), regularity index (RI), and coupling index (CP) *x*-axes] are presented for the six considered AP phenotypes. Top and bottom rows show results for left atrium (LA) and right atrium (RA), under partial block of I_K1_ (left column), I_NaK_ (middle column), and I_Na_ (right column), respectively.

Percentual variations of the analyzed indices following the three ionic blocks with respect to the averaged values in the six virtual phenotypes are shown in Figure 8 for both the LA (top panels) and the RA (bottom panels). Bars of the same color in Figure 8 represent each virtual phenotype, whereas the analyzed indices are shown in the horizontal axis. The effects of these ionic blocks were qualitatively similar between virtual phenotypes, except for some particular cases. For example, the block of any of the three ionic currents promoted interatrial differences in RI and CP in the short APD_90_ phenotype by increasing both indices in the LA and decreasing them in the RA. The opposite occurred in the short APD_20_ phenotype, in which RI and CP were decreased in the LA and increased in the RA.

## Discussion

In this study, six 3D human atrial models with different AP phenotypes were constructed to quantify differences in patterns, regularity, and organization of AF dynamics in control and following ionic inhibition. The AF dynamics in the human atrial models are shown to yield quantified indices in agreement with those obtained from AF patients in the Ann Arbor database. This supports the credibility of human atrial models to be used as test beds for antiarrhythmic therapy. Through analysis of fibrillatory electrical activation in the six human atrial models, we provide novel findings on patterns of human atria fibrillatory dynamics in different electrophysiological phenotypes and their response to ionic current block.

In our simulations, human atria with long vs short APD_90_ yielded significantly different fibrillatory patterns, with clear differences in DF and RI. Longer APD_90_ was associated with slower and more regular fibrillatory patterns. This is in good agreement with the close correlation between APD_90_ and effective refractory period (ERP) and their strong effects on tissue wavelength, which determines reentrant dynamics.

Importantly, differences in APD_50_ and APD_20_ also highly influenced the propagation patterns. Long APD_50_ resulted in interatrial conduction block, and this led to significant interatrial differences in DF and OI. Long APD_20_ phenotype also led to interatrial propagation block of the reentrant circuits, resulting in self-termination of the arrhythmia. This suggests that therapies aiming at prolonging the early phase of repolarization might constitute novel antiarrhythmic strategies for the pharmacological management of AF. Simulations also show that virtual phenotypes with short atrial AP present rapid reentrant rotors, highly stable in the LA, and more irregular in the RA, which is larger and, therefore, allows coexistence of a higher number of wavelets and wave meandering. For all AP phenotypes investigated, electrical activation in the LA is generally faster and more organized than in the RA, irrespective of specific AP differences considered.

The selective ion channel inhibitions considered in this study yielded similar propagation patterns qualitatively consistent between virtual AP phenotypes: inhibition of I_K1_, I_NaK_, or I_Na_ entailed a more notable wave meandering that reduces the organization of the arrhythmia for all the AP phenotypes, either by slowing down electrical propagation in the human atria (I_Na_ block) or enlarging the propagation wavefront (I_K1_ and I_NaK_ block). The results are consistent with previous studies (Murgatroyd and Camm, 1993; Kneller et al., 2005; Pandit et al., 2005; Noujaim et al., 2007; Comtois et al., 2008), lending further credibility to the simulation results. Our study, therefore, illustrates how human whole-atria models provide a test-bed in which to test antiarrhythmic strategies, importantly accounting for phenotypic differences in atrial electrophysiological as observed across human individuals.

Our simulations also show that I_K1_ inhibition had a more prominent antiarrhythmic decrease effect in DF than I_NaK_ or I_Na_ reduction, which is explained by the importance of I_K1_ in ERP modulation in human atrial cardiomyocytes. Block of I_K1_ was also proven to be more effective to terminate AF than block of I_Na_ in the sheep heart (Filgueiras-Rama et al., 2012). Furthermore, in mouse hearts, upregulation of I_K1_ increased velocity and stability of reentrant rotors (Noujaim et al., 2007) and downregulation of I_K1_ was effective to terminate some arrhythmias (Noujaim et al., 2011).

Furthermore, our simulation results indicate larger disorganizations of reentry (reduced OI) for short APD_50_ and APD_20_ phenotypes in response to any of the three interventions compared to the effects of these interventions in the other APD phenotypes. Such a reduced organization has been previously related with less stable reentrant circuits (antiarrhythmic), but paradoxically with a larger number of secondary wavelets (pro-arrhythmic) (Everett et al., 2001). This suggests the importance of different electrophysiological substrates and early repolarization in determining the response to drug therapy.

Activation patterns become more irregular with inhibition of I_K1_, I_NaK_, or I_Na_ in the simulations, and CP between pairs of adjacent EGMs became weaker after ionic current block. This is explained by increased wave meandering and, therefore, decreased morphological similarity between adjacent atrial EGMs. Wave meandering was more pronounced in the RA than in the LA due to its larger size and the larger distance to the ectopic foci near the PV in our simulations. Our simulation results are in agreement with previous studies showing decreased stability of rotors in the human atria due to I_K1_ and I_Na_ inhibition (Kneller et al., 2005; Ehrlich and Nattel, 2009; Pandit and Jalife, 2013) as well as variable efficacy of these pharmacological interventions in the conversion of AF, likely due to differences in underlying electrophysiological substrates.

I_Na_ inhibition effectively slowed CV in both LA and RA for the six virtual phenotypes, increased wavelength of reentrant circuits *via* strong prolongation of ERP, and reduced DF in the whole atria for all AP phenotypes, as previously established in both computational and experimental studies (Pandit et al., 2005; Burashnikov et al., 2007; Antzelevitch and Burashnikov, 2010; Sánchez et al., 2012). However, the possible side effects of sodium channel blockers on ventricular electrophysiology highlight the necessity of finding more effective atrial-selective therapies.

Finally, I_NaK_ inhibition in the human virtual atrial models, with the exception of the long APD_90_ phenotype in which the arrhythmia terminated, led to an overall less notable modulation of fibrillatory dynamics and organization indices than those associated to I_K1_ or I_Na_, regardless of the atrial AP phenotype. This was in spite of evidence supporting the role of the NaK pump in modulating atrial APD and its rate adaptation (Grandi et al., 2011; Koivumäki et al., 2011; Sánchez et al., 2012; Bueno-Orovio et al., 2013), as well as the long standing use of digoxin in the cardio-conversion of AF (Lévy, 1997). The slow adaptation of APD secondary to I_NaK_ block could, therefore, entail important contributions to the efficacy of pharmacological inhibition of the NaK pump (Sánchez et al., 2012). However, these would take place at time scales much longer than the acute inhibitions considered here for reasons related to computational tractability of whole atria simulation studies at the whole-atrial level.

### Limitations

In this study, the Maleckar et al. model (Maleckar et al., 2009) was used to simulate cellular electrophysiology, given its improved representation of human atrial repolarization dynamics. The choice of a different human atrial cell model could make results be different from those shown in this study. Nevertheless, AP phenotypes in AF with other human atrial cell models (Courtemanche et al., 1998; Grandi et al., 2011) after experimental calibration were shown to be similar to those with the Maleckar et al. model in a previous study (Sánchez et al., 2014).

Reentrant behavior was generated using a particular stimulation protocol consisting of periodic stimuli applied at the SAN followed by six extra-stimuli near the RPV. However, AF in clinical practice may present multiple ectopic foci that trigger and sustain the arrhythmia, and their location may be variable as well (Jalife et al., 2002). Furthermore, a healthy atrial anatomical model was used in this study, in order to focus on the effects of AF-induced electrophysiological rather than structural remodeling (like fibrosis), which would also affect arrhythmia generation and maintenance (Burstein and Nattel, 2008).

The effects of selective ionic blocks were analyzed individually in this study. However, many antiarrhythmic drugs, such as flecainide, digitalis, or barium, have multiple ionic targets and their simultaneous inhibitions could have synergistic and/or non-linear effects on arrhythmic markers. Therefore, a multi-target approach like that used in Sivagangabalan et al. (2014) could be interesting to explore in future studies. In addition, we only simulated moderate selective ion channel inhibitions (30% I_K1_ block, 30% I_NaK_ block, and 15% I_Na_ block). Larger ion channel blocks might lead to a successful termination of atrial fibrillatory dynamics in more virtual patients, but they would also have stronger effects in the ventricles, raising potential safety concerns.

The relatively small number of virtual electrodes used (49) led to visible interpolation artifacts in the figures. Nevertheless, this precision was high enough to allow proper characterization of arrhythmic behavior, since the electrical propagation wavefronts were much wider than the separation between adjacent electrodes.

As a final remark, the random assignment of a specific model within each subpopulation to each cell in the 3D model could potentially lead to neighboring cells with very different electrophysiological properties. This effect was, however, reduced for two reasons: (1) variability in the AP within each subpopulation was relatively small (first and fourth quartiles of the subpopulations were chosen to represent the slight variability present within one subject); (2) the electrotonic coupling between cells strongly smoothed electrical gradients during propagation (Figure S1 in Supplementary Material).

### Conclusion

Inter-patient variability in cell repolarization entails notable differences in the organization and stability of arrhythmias in AF-electrophysiologically-remodeled tissue. Specific ionic blocks of I_K1_, I_NaK_, and I_Na_ reduce the regularity of fibrillation patterns and promote interatrial differences, especially for the phenotypes with short APD at 20, 50, and/or 90% repolarization.

## Author Contributions

All authors equally contributed to the conception of the work, revising it critically for important intellectual content, and final approval of the version to be published, ensuring that questions related to the accuracy or integrity of any part of the work were appropriately investigated and resolved. EP and BR acted as supervisors of the research, AB-O mainly contributed in methodological aspects, and CS was responsible for conducting all the simulations, analysis of results, and drafting the work.

## Conflict of Interest Statement

The authors declare that the research was conducted in the absence of any commercial or financial relationships that could be construed as a potential conflict of interest.
