# Supplementary material for: Atrial Fibrillation Dynamics and Ionic Block Effects in Six Heterogeneous Human 3D Virtual Atria with Distinct Repolarization Dynamics
Source: Front Bioeng Biotechnol. 2017 May 8;5:29. doi: 10.3389/fbioe.2017.00029 (PMC5420585; doi:10.3389/fbioe.2017.00029)
Supplement: Supplementary file 1 [file Data_Sheet_1.DOCX]

Supplementary Material

Atrial Fibrillation Dynamics and Ionic Block Effects in Six Heterogeneous Human 3D Virtual Atria with Distinct Repolarization Dynamics

Carlos Sánchez*, Alfonso Bueno-Orovio, Esther Pueyo, Blanca Rodríguez

*** Correspondence:** Corresponding Author: cstapia@unizar.es

**Supplementary Table 1** shows the specific values of conduction velocity (CV), anisotropic ratio (transversal to longitudinal ratio of conductivity) and particular modulation of four ionic currents (I_to_, I_CaL_, I_Kr_ and I_KACh_) in the different regions of the atria to account for spatial heterogeneities, in agreement with previous studies (Atienza et al., 2006; Krueger et al., 2011; Seemann et al., 2006; Tobon et al., 2013).

Electrophysiological heterogeneities between LA and RA were included through gradients in I_Kr_ (1.6 times larger in LA than RA) and I_KACh_ (2 times larger in LA than RA) as shown in **Supplementary Table 1**, based on (Atienza et al., 2006). **Supplementary Figure** **1** shows distributions of APD_90_, APD_50_ and APD_20_ in the six virtual phenotypes and histograms for each APD distribution with separation between LA and RA. It can be observed that the regional differences in the atria described in **Supplementary Table 1** have stronger effects on repolarization than cell-to-cell differences.

# Classification of rotors and reentrant circuits

**Supplementary Table 2** shows the complete list of rotors and reentrant circuits, i.e. those without phase singularities but rotating around an anatomical structure denoted by “Anat. Reent.” in the Table, in the LA and the RA for each simulation. The rotors and reentrant circuits are classified according to their duration (left columns) in three groups: < 2 s, 2 – 5 s, and > 5 s, and their pathlength (right columns), measured as the maximum distance covered by the phase singularity of a rotor, also in three groups: < 1 cm, 1 – 2 cm, and > 2 cm. In the simulations with more than one rotor in the same cavity (LA or RA) and different durations and pathlengths, rotors are written in bold and italics for their identification.

# Effects of Acetylcholine on measurable indices in the atria

**Supplementary Figure 2** shows the values of the measurable atrial indices considered in this study: dominant frequency (DF, **Supplementary Figure 2A**), organization index (OI, (**Supplementary Figure 2B**), regularity index (RI, **Supplementary Figure 2C**), and coupling index (CP, **Supplementary Figure 2D**) for simulations with two levels of Acetylcholine ([ACh]): 1 nM and 5 nM. Despite showing slightly smaller DF values, the simulation with [ACh] = 5 nM presented a more chaotic activation pattern than that with [ACh] = 1 nM, reflected on the reduced values of RI, OI and CP.

# Arrhythmia-related indices computation

**Supplementary Figure** **3** shows an example of how arrhythmia-related indices reflect electrical propagation through the atria. Results correspond to the Maleckar et al. model in chronic-AF conditions (i.e. 70% decrease in I_CaL_, 50% decrease in I_to_, 50% decrease in I_Kur_, and 100% increase in I_K1_). Computation of the indices for the analysis of fibrillatory dynamics was performed as follows:

• Dominant frequency (DF) was obtained as the location of the maximum peak of the power spectral density of the post-processed EGMs. High values of DF are related to arrhythmic fast activating tissue. Heterogeneous distribution of DF in the atria is usually related to local propagation blocks.

• Organization index (OI) was calculated from the power spectral density as the ratio of the sum of areas under the DF±0.75 Hz and its first three harmonics (2DF±0.75 Hz, 3DF±0.75 Hz, and 4DF±0.75 Hz) over the total area under the power spectral density (Everett et al., 2001). Low values of OI are associated with irregular non-periodic activation sequences (see **Supplementary Figure 2**).

• Regularity index (RI) and coupling index (CP) were calculated using morphological comparisons between local activation waves (LAWs, x_i_) in every EGM. The central points of these LAWs were obtained as the times were the steepest deflections occurred, and their durations for posterior analysis were set to 90 ms (±45 ms from the central point) in order to avoid more than one activation in the same LAW. Two normalized LAWs ($\hat{x}$_i_) were considered similar if the accumulated difference between them (d($\hat{x}$_i_,$\hat{x}$_j_)) was lower than a certain threshold (ε), as shown in the following equations from (Faes and Ravelli, 2007):

$\boldsymbol{RI}_{\boldsymbol{x}}\boldsymbol{=}\frac{\boldsymbol{N}_{\boldsymbol{S}}}{\boldsymbol{N}_{\boldsymbol{tot}}}\boldsymbol{=}\frac{\sum_{\boldsymbol{i=1}}^{\boldsymbol{N}} \sum_{\boldsymbol{j=1;j\neq i}}^{\boldsymbol{N}} \boldsymbol{\Theta}\left( \boldsymbol{\varepsilon-d}\left( \hat{\boldsymbol{x}_{\boldsymbol{i}}}\boldsymbol{,}\hat{\boldsymbol{x}_{\boldsymbol{j}}} \right) \right)}{\boldsymbol{N}\left( \boldsymbol{N-1} \right)}$ (1)

$\boldsymbol{\rho}_{\boldsymbol{xy}}\boldsymbol{=}\frac{\boldsymbol{N}_{\boldsymbol{S}_{\boldsymbol{xy}}}}{\boldsymbol{N}_{\boldsymbol{tot}_{\boldsymbol{xy}}}}\boldsymbol{=}\frac{\sum_{\boldsymbol{i=1}}^{\boldsymbol{N}} \sum_{\boldsymbol{j=1;j\neq i}}^{\boldsymbol{N}} \boldsymbol{\Theta}\left( \boldsymbol{\varepsilon-max}\left\{ \boldsymbol{d}\left( \hat{\boldsymbol{x}_{\boldsymbol{i}}}\boldsymbol{,}\hat{\boldsymbol{x}_{\boldsymbol{j}}} \right)\boldsymbol{, d}\left( \hat{\boldsymbol{y}_{\boldsymbol{i}}}\boldsymbol{,}\hat{\boldsymbol{y}_{\boldsymbol{j}}} \right) \right\} \right)}{\boldsymbol{N}\left( \boldsymbol{N-1} \right)}$ (2)

$\boldsymbol{CP}_{\boldsymbol{xy}}\boldsymbol{=}\frac{\boldsymbol{\rho}_{\boldsymbol{xy}}}{\boldsymbol{RI}_{\boldsymbol{x}}\boldsymbol{+}\boldsymbol{RI}_{\boldsymbol{y}}}$ (3)

Interpretation of RI is similar to that of OI, but RI analysis is performed in the time domain, whereas OI information is extracted from the frequency domain. High values of RI reflect stabilized activation sequences over time, whereas high values of CP are associated with similar activation sequences in neighboring regions. **Supplementary Figure 3** shows low RI in a couple of small areas where some propagation wavefronts are blocked or collide with other wavefronts. Propagation patterns changing over time in the RA entails low values of CP as compared to LA (**Supplementary Figure 3**).

# Validation of simulation results by comparison to EGM database

**Supplementary Figure 4A** shows values of EGM-derived indices (DF, OI and RI) as calculated in each of the EGMs registered at the RA in AF patients from the Ann Arbor database. DF values obtained in these EGMs were between 3.84 and 6.56 Hz (**Supplementary Figure 4B**), in good agreement with DF values from simulations in all six models (**Figure 4**). Similarly, experimental OI (from 0.27 to 0.89) and RI (from 0.4 to 1) values, shown in **Supplementary Figure 4B**, were in agreement with those obtained in the six virtual models (**Figure 4**).

# Numerical convergence of the computational simulations

**Supplementary Figure 5** shows electrograms measured at virtual electrodes on the RA (panels (**A**) and (**B**)) and on the LA (panels (**C**) and (**D**)) for two simulations during the preconditioning stage of the simulations, i.e. with periodic stimulation, and different computational time-steps: 0.02 ms (blue) and 0.04 ms (red). As shown in the Figure, the electrograms are practically equal during the simulation for both time-steps, ensuring convergence of the numerical results of this study.

**References**

Atienza, F., Almendral, J., Moreno, J., Vaidyanathan, R., Talkachou, A., Kalifa, J., et al. (2006). Activation of inward rectifier potassium channels accelerates atrial fibrillation in humans: evidence for a reentrant mechanism. *Circulation* 114, 2434–2442. doi:10.1161/CIRCULATIONAHA.106.633735.

Everett, T. H., 4th, Kok, L. C., Vaughn, R. H., Moorman, J. R., and Haines, D. E. (2001). Frequency domain algorithm for quantifying atrial fibrillation organization to increase defibrillation efficacy. *IEEE Trans. Biomed. Eng.* 48, 969–978.

Faes, L., and Ravelli, F. (2007). A morphology-based approach to the evaluation of atrial fibrillation organization. *IEEE Eng. Med. Biol. Mag. Q. Mag. Eng. Med. Biol. Soc.* 26, 59–67.

Krueger, M. W., Schmidt, V., Tobón, C., Weber, F. M., Lorenz, C., Keller, D. U. J., et al. (2011). “Modeling Atrial Fiber Orientation in Patient-Specific Geometries: A Semi-automatic Rule-Based Approach,” in *Functional Imaging and Modeling of the Heart* Lecture Notes in Computer Science., eds. D. N. Metaxas and L. Axel (Springer Berlin Heidelberg), 223–232.

Seemann, G., Höper, C., Sachse, F. B., Dössel, O., Holden, A. V., and Zhang, H. (2006). Heterogeneous three-dimensional anatomical and electrophysiological model of human atria. *Philos. Transact. A Math. Phys. Eng. Sci.* 364, 1465–1481. doi:10.1098/rsta.2006.1781.

Tobon, C., Ruiz-Villa, C. A., Heidenreich, E., Romero, L., Hornero, F., and Saiz, J. (2013). A three-dimensional human atrial model with fiber orientation. Electrograms and arrhythmic activation patterns relationship. *PloS One* 8, e50883. doi:10.1371/journal.pone.0050883.

**Supplementary Table 1.** Tissue anisotropy and heterogeneities.

|  | Anisotropic ratio | CV (cm/s) | *I_to_** | *I_CaL_** | *I_Kr_** | *I_KACh_** |
| --- | --- | --- | --- | --- | --- | --- |
| LA | 1:2 | 59 | 1 | 1 | √1.6 | √2 |
| RA | 1:2 | 59 | 1 | 1 | 1/√1.6 | 1/√2 |
| SAN | 1:1 | 31 | 1 | 1 | 1 | 1 |
| CT | 1:10 | 116 | 1.35 | 1.6 | 0.9 | 1 |
| PM | 1:2 | 98 | 1.05 | 0.95 | 0.9 | 1 |
| FO | 1:2 | 59 | 1 | 1 | 1 | 1 |
| RAPG | 1:2 | 59 | 1 | 1 | 1 | 1 |
| IAB | 1:2 | 59 | 1 | 1 | 1 | 1 |
| BB | 1:2 | 98 | 1 | 1 | 1 | 1 |
| CTI | 1:1 | 44 | 1 | 1 | 1 | 1 |
| LAPG | 1:2 | 59 | 0.65 | 1.05 | 2.75 | 1 |
| AVR | 1:2 | 59 | 1.05 | 0.65 | 3 | 1 |

**Multiplicative factors with respect to the default values in the model*

**Supplementary Table 2.** Duration and pathlength of rotors and reentrant circuits

|  | **Duration** | | | **Pathlength** | | |
| --- | --- | --- | --- | --- | --- | --- |
|  | **<2 s** | **2-5 s** | **>5 s** | **<1cm** | **1 - 2 cm** | **>2 cm** |
| **(Default)** |  |  |  |  |  |  |
| longAPD_90_ |  |  | 1 LA&RA  (Anat. Reent.  LA-RA) |  |  |  |
| shortAPD_90_ | 2 RA | 1 RA | 1 LA; 1 LA  (Anat. Reent.  RPV) | 1 LA | 3 RA |  |
| longAPD_50_ |  |  | 1 LA |  | 1 LA |  |
| shortAPD_50_ |  | 1 RA | ***1 RA;*** 1 LA |  | 1 RA; 1 LA | ***1 RA*** |
| longAPD_20_ |  |  | 1 LA&RA  (Anat. Reent.  LA-RA)  (self-terminated  after 6.3 s) |  |  |  |
| shortAPD_20_ | 2 RA | ***1 RA*** | ***1 RA;*** 1 LA |  | 2 RA; 1 LA | ***2 RA*** |
| **(*I_K1_* block)** |  |  |  |  |  |  |
| longAPD_90_ |  |  | 1 RA  (Anat. Reent.  RA) |  |  |  |
| shortAPD_90_ |  | 2 RA | 1 LA | 1 LA |  | 2 RA |
| longAPD_50_ |  |  | 1 LA&RA  (Anat. Reent.  AV ring) |  |  |  |
| shortAPD_50_ | 1 RA | ***1 RA*** | 1 LA  (Anat. Reent.  AV ring) | 1 RA |  | ***1 RA*** |
| longAPD_20_ |  |  | 1 LA; 1 LA  (Anat. Reent.  AV ring) | 1 LA |  |  |
| shortAPD_20_ |  |  | 1 LA  (Anat. Reent.  AV ring) |  |  |  |
| **(*I_Na_* block)** |  |  |  |  |  |  |
| longAPD_90_ |  |  | 1 RA  (Anat. Reent.  RA) |  |  |  |
| shortAPD_90_ |  | 1 RA | ***1 RA;*** 1 LA | ***1 RA;*** 1 LA | 1 RA |  |
| longAPD_50_ | 5 RA | 1 RA |  | 4 RA | 1 RA | 1 RA |
| shortAPD_50_ | 1 RA |  | 1 RA; 1 LA  (Anat. Reent.  RPV) |  |  | 2 RA |
| longAPD_20_ | 1 RA | 1 RA | 1 LA | 1 LA | 2 RA |  |
| shortAPD_20_ | 3 LA | 1 RA | 1 LA  (Anat. Reent.  RPV) | 3 LA | 1 RA |  |
| **(*I_NaK_* block)** |  |  |  |  |  |  |
| longAPD_90_ |  |  | Arrhythmia  abolished |  |  |  |
| shortAPD_90_ | 5 RA |  | 1 LA | 1 RA; 1 LA | 3 RA | 1 RA |
| longAPD_50_ | 1 LA | 2 RA | 1 LA  (Anat. Reent.  LPV) | 1 LA | 2 RA |  |
| shortAPD_50_ | 3 LA |  | 1 LA  (Anat. Reent.  RPV) | 3 LA |  |  |
| longAPD_20_ | 1 LA  (Anat. Reentry  LA) | 2 LA |  |  | 2 LA |  |
| shortAPD_20_ | 5 LA |  | 1 LA  (Anat. Reent.  RPV) | 1 LA | 4 LA |  |
|  |  |  |  |  |  |  |

**
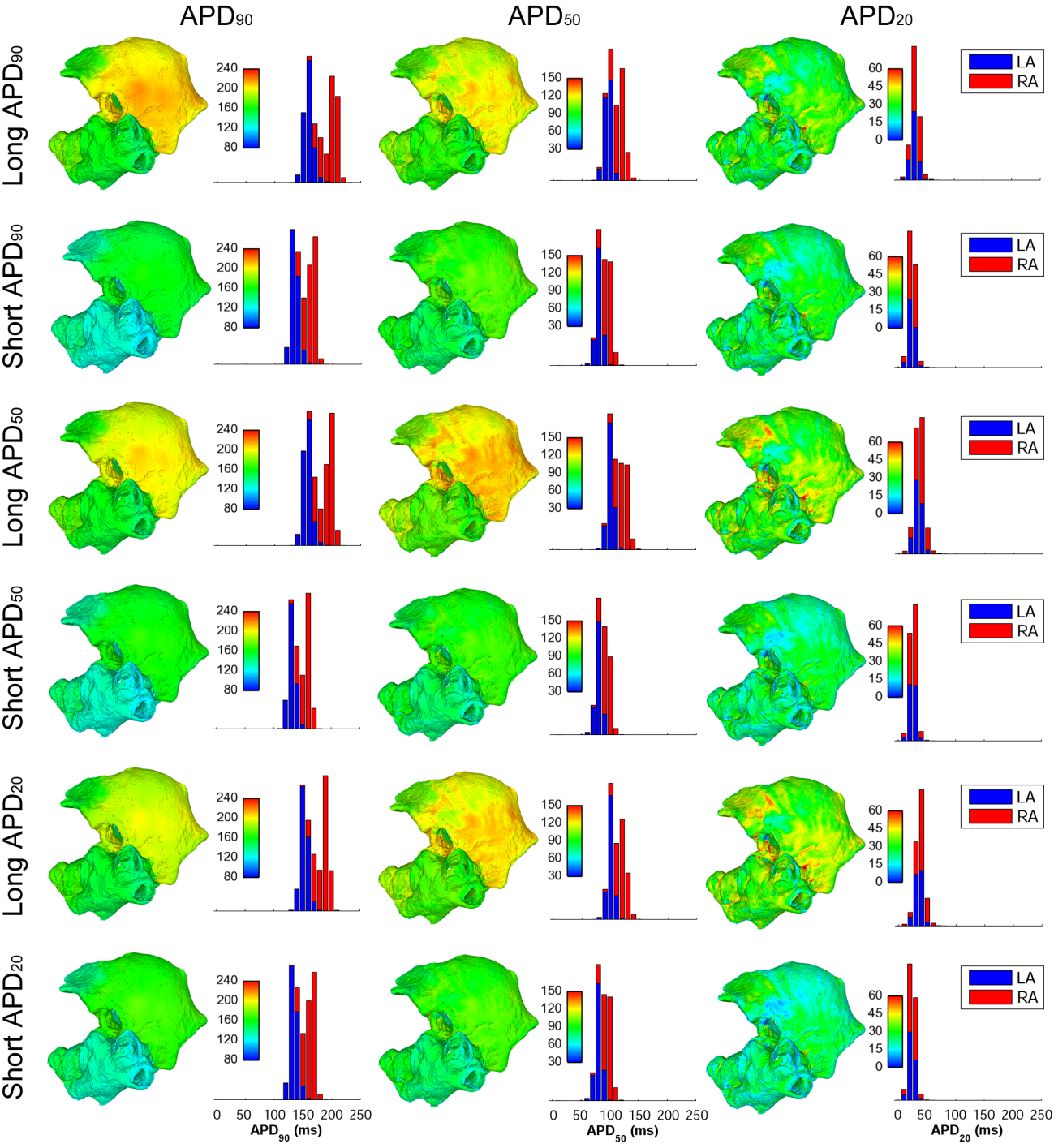
**

**Supplementary Figure 1.** Distributions of APD_90_ (left), APD_50_ (middle), APD_20_ (right) in the six virtual phenotypes (from top to bottom: Long APD_90_, Short APD_90_, Long APD_50_, Short APD_50_, Long APD_20_, and Short APD_20_). Histograms for each APD distribution with separation between LA (blue) and RA (red).


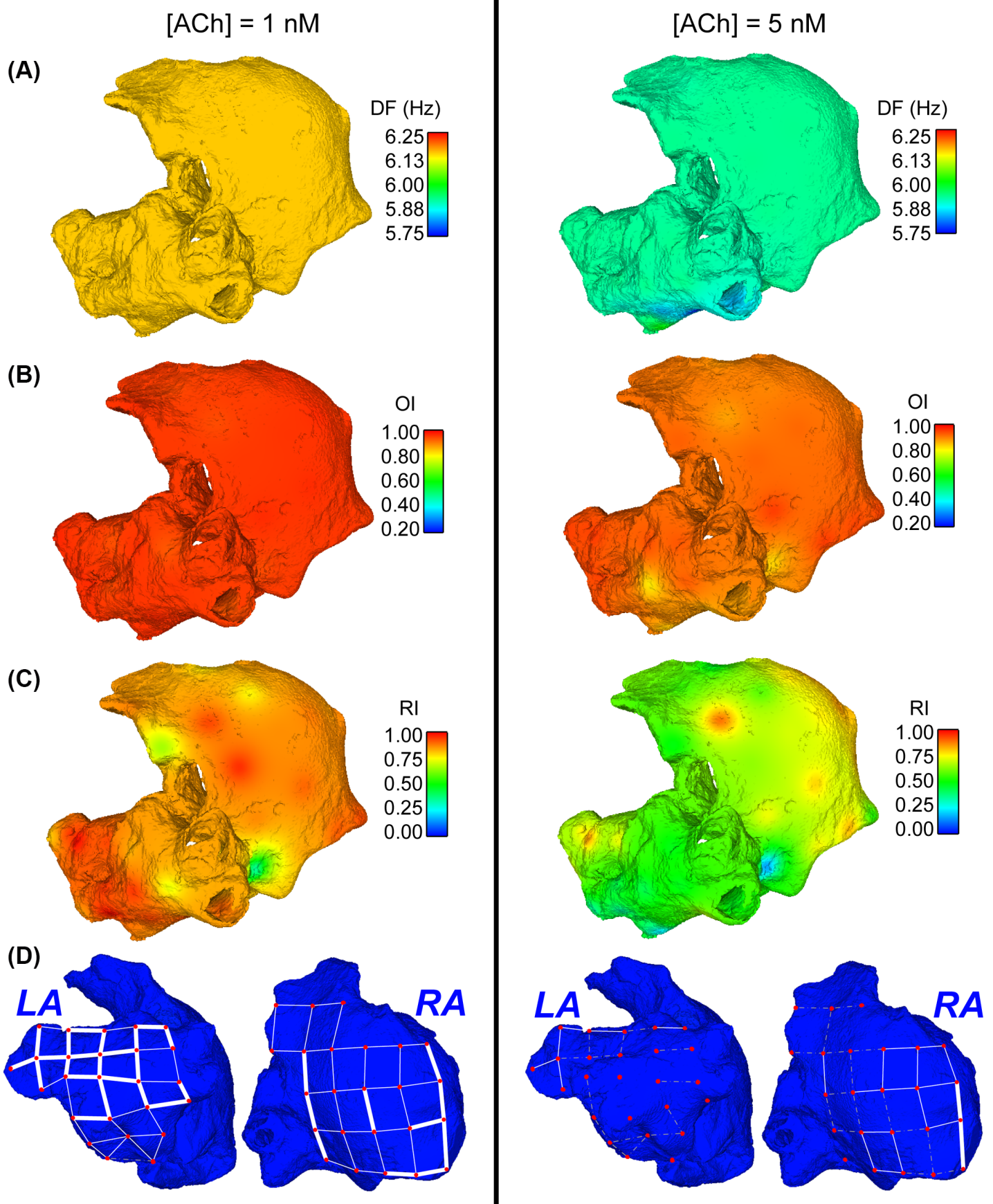


**Supplementary Figure 2.** Dorsal view of the atria for the chronic-AF simulations with [ACh]=1 nM (left) and 5 nM (right), respectively. Corresponding measurable indices are shown: interpolated maps of DF (**A**), OI (**B**) and RI (**C**), and CP (**D**) between adjacent EGMs (thick white lines represent CP>0.9, thin solid lines represent 0.75≤CP<0.9 and soft gray lines represent 0.6≤CP<0.75. CP<0.6 is represented by absence of lines).

**
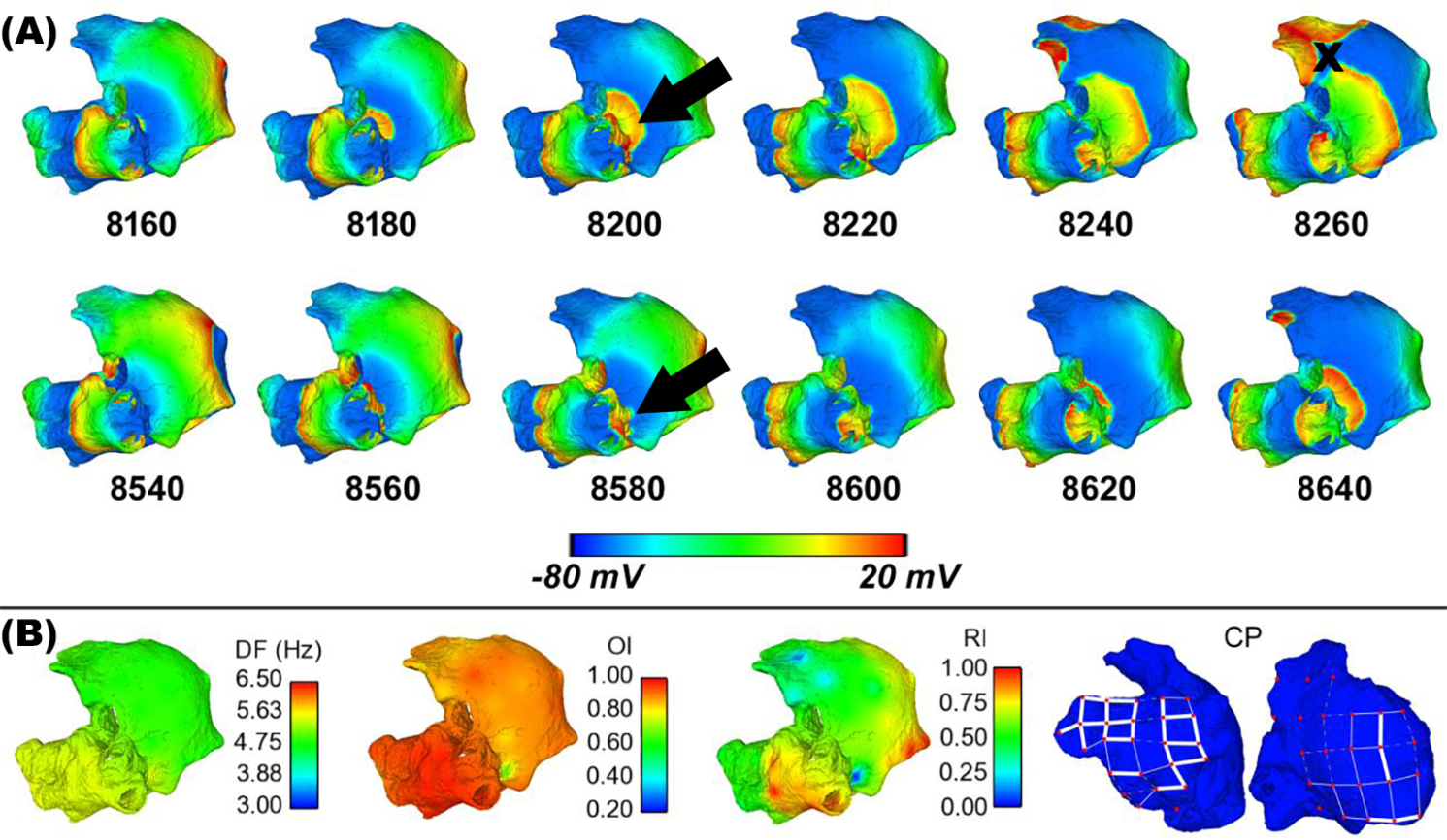
**

**Supplementary Figure 3.** Dorsal view of the atria for the default Maleckar et al. model in chronic-AF. **(A):** Snapshots of the transmembrane potential at the time instants shown below in bold (numbers in ms). Arrows in snapshots 8200 and 8580 indicate an area with low values of RI and OI due to transient propagation blocks. ‘X’ in snapshot 8260 marks a wavefront collision area associated with low RI values. **(B):** Corresponding measurable indices (interpolated maps of DF, OI and RI, and CP between adjacent EGMs (thick white lines represent CP>0.9, thin solid lines represent 0.75≤CP<0.9 and soft gray lines represent 0.6≤CP<0.75. CP<0.6 is represented by absence of lines)) for the simulation shown in panel **(A)**.

**
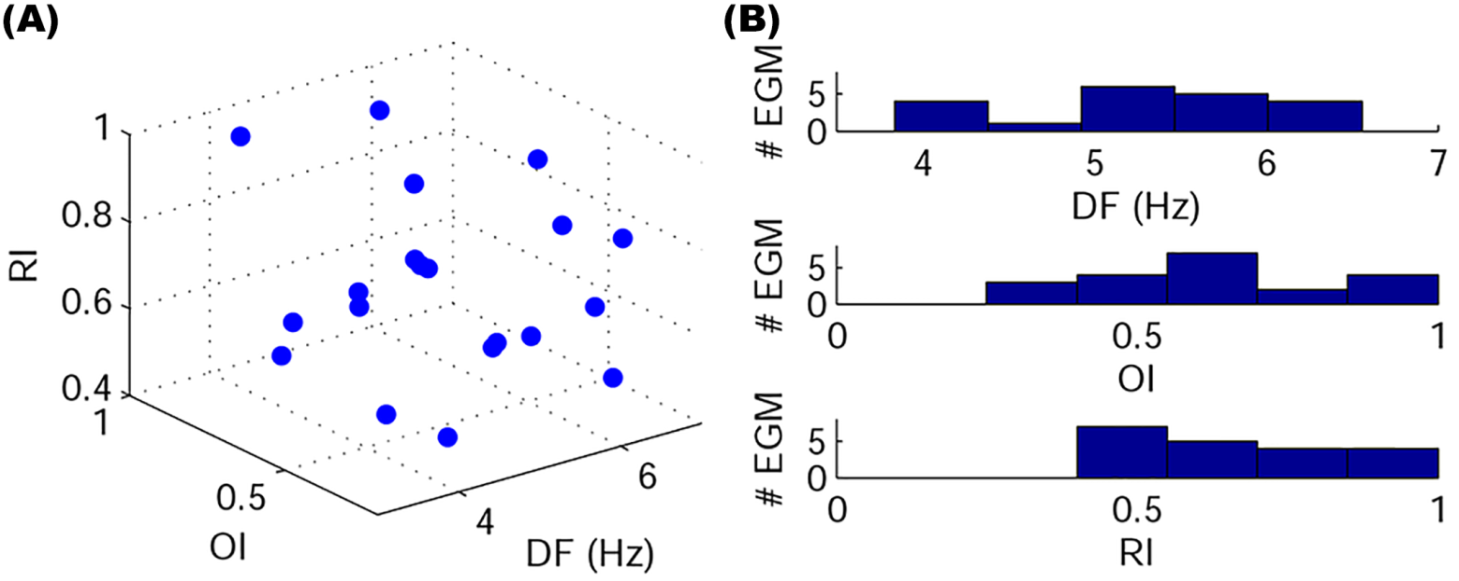
**

**Supplementary Figure 4. (A):** Scatter plot of DF, OI and RI calculated from the Ann Arbor database. **(B):** Histograms showing the number of EGM for different intervals of DF, OI and RI.

**
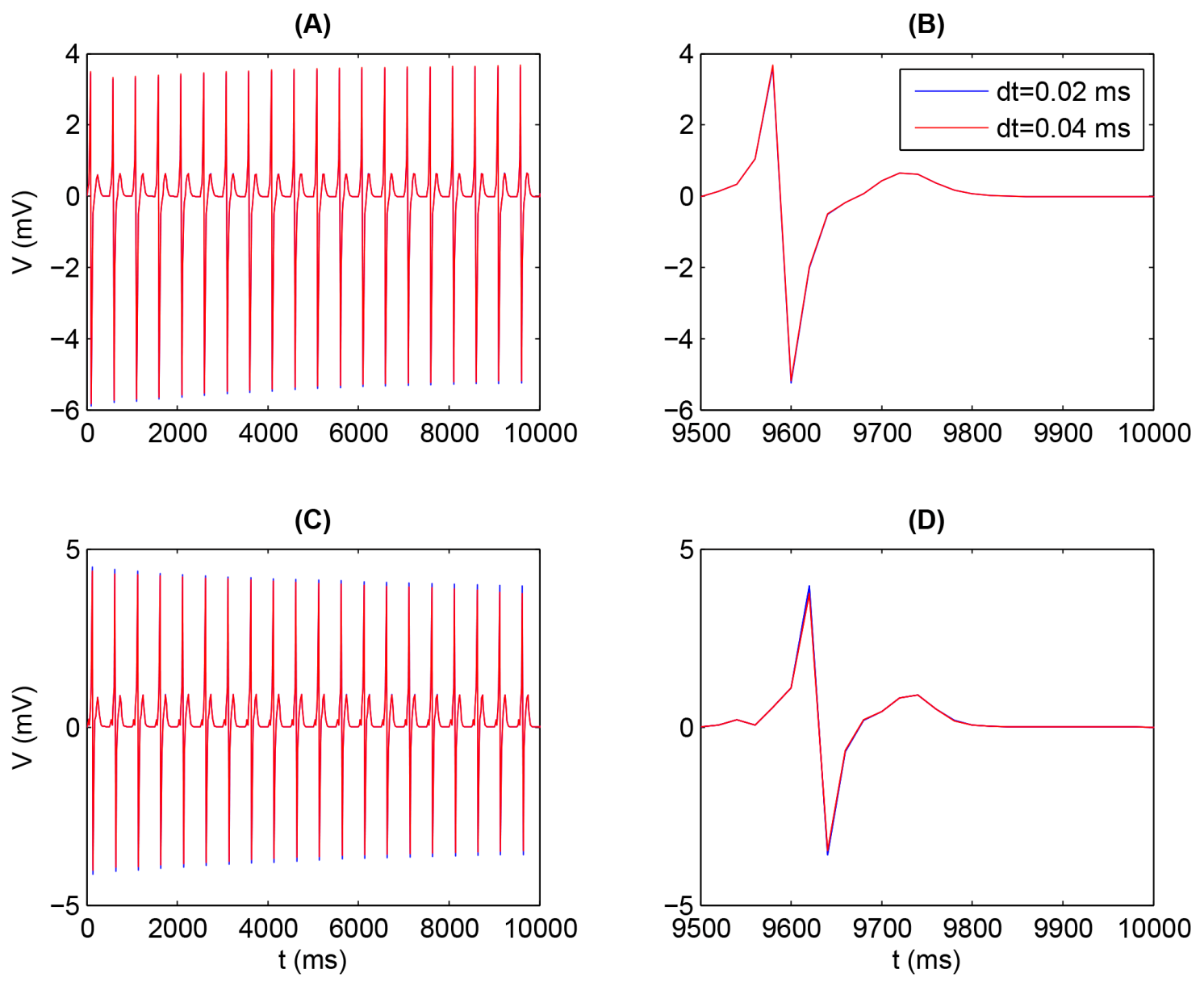
**

**Supplementary Figure 5. (A):** Electrogram measured at a virtual electrode on the RA for two simulations with periodic stimulation and different computational time-steps: 0.02 ms (blue) and 0.04 ms (red). **(B):** Last beat of panel **(A)**. **(C)**: Electrogram measured at a virtual electrode on the LA for two simulations with periodic stimulation and different computational time-steps: 0.02 ms (blue) and 0.04 ms (red). **(D):** Last beat of panel **(C)**.
